# Enhancing accessibility for improved diagnosis with modified EfficientNetV2-S and cyclic learning rate strategy in women with disabilities and breast cancer

**DOI:** 10.3389/fmed.2024.1373244

**Published:** 2024-03-07

**Authors:** Moteeb Al Moteri, T. R. Mahesh, Arastu Thakur, V. Vinoth Kumar, Surbhi Bhatia Khan, Mohammed Alojail

**Affiliations:** ^1^Department of Management Information Systems, College of Business Administration, King Saud University, Riyadh, Saudi Arabia; ^2^Department of Computer Science and Engineering, Faculty of Engineering and Technology, JAIN (Deemed-to-be University), Bangalore, India; ^3^School of Computer Science Engineering and Information Systems, Vellore Institute of Technology, Vellore, India; ^4^Department of Data Science, School of Science Engineering and Environment, University of Salford, Manchester, United Kingdom; ^5^Department of Electrical and Computer Engineering, Lebanese American University, Byblos, Lebanon

**Keywords:** machine learning, EfficientNetV2, histopathological image classification, deep learning, image processing, BreakHis dataset, individuals with disabilities, medical image interpretation

## Abstract

Breast cancer, a prevalent cancer among women worldwide, necessitates precise and prompt detection for successful treatment. While conventional histopathological examination is the benchmark, it is a lengthy process and prone to variations among different observers. Employing machine learning to automate the diagnosis of breast cancer presents a viable option, striving to improve both precision and speed. Previous studies have primarily focused on applying various machine learning and deep learning models for the classification of breast cancer images. These methodologies leverage convolutional neural networks (CNNs) and other advanced algorithms to differentiate between benign and malignant tumors from histopathological images. Current models, despite their potential, encounter obstacles related to generalizability, computational performance, and managing datasets with imbalances. Additionally, a significant number of these models do not possess the requisite transparency and interpretability, which are vital for medical diagnostic purposes. To address these limitations, our study introduces an advanced machine learning model based on EfficientNetV2. This model incorporates state-of-the-art techniques in image processing and neural network architecture, aiming to improve accuracy, efficiency, and robustness in classification. We employed the EfficientNetV2 model, fine-tuned for the specific task of breast cancer image classification. Our model underwent rigorous training and validation using the BreakHis dataset, which includes diverse histopathological images. Advanced data preprocessing, augmentation techniques, and a cyclical learning rate strategy were implemented to enhance model performance. The introduced model exhibited remarkable efficacy, attaining an accuracy rate of 99.68%, balanced precision and recall as indicated by a significant F1 score, and a considerable Cohen’s Kappa value. These indicators highlight the model’s proficiency in correctly categorizing histopathological images, surpassing current techniques in reliability and effectiveness. The research emphasizes improved accessibility, catering to individuals with disabilities and the elderly. By enhancing visual representation and interpretability, the proposed approach aims to make strides in inclusive medical image interpretation, ensuring equitable access to diagnostic information.

## 1 Introduction

Breast cancer remains a widespread and serious health issue worldwide, posing significant challenges for healthcare professionals and researchers. The intricate nature of this disease demands innovative approaches to diagnosis and treatment, with histopathological image analysis emerging as a crucial tool in this endeavor. The accurate interpretation and classification of these images are paramount for delivering timely and effective medical care to breast cancer patients. Against this backdrop, our research endeavors to elevate the accuracy and reliability of breast cancer histopathological image classification, aiming to make substantial contributions to the advancement of diagnostic and therapeutic practices.

To understand the significance of histopathological image analysis in breast cancer, it is essential to delve into the historical context of breast cancer diagnosis. Over the years, there has been a paradigm shift from traditional diagnostic methods to more sophisticated and precise techniques. Initially, clinical evaluation and palpation were the primary means of detecting breast abnormalities. However, the limitations of these methods in terms of sensitivity and specificity led to the exploration of imaging modalities such as mammography.

The introduction of mammography significantly transformed breast cancer screening, allowing for the observation of internal structures within the breast. While effective in identifying tumors at early stages, mammography has limitations, such as the occurrence of false positives and patient discomfort. With technological advancements, other imaging methods like ultrasound and magnetic resonance imaging (MRI) have been integrated into diagnostic processes, providing supplementary data to enhance diagnostic precision. Histopathological examination of breast tissue samples remains the gold standard for confirming the presence of cancer and determining its characteristics. When a suspicious lesion is detected through imaging, a biopsy is often performed, and the excised tissue undergoes histopathological analysis. This process involves the examination of cellular and tissue morphology under a microscope, allowing pathologists to identify cancerous cells, assess tumor grade, and characterize the extent of invasion.

In recent years, the integration of digital pathology and computer-aided diagnostic tools has transformed histopathological image analysis. Digital pathology involves the digitization of glass slides to create high-resolution digital images that can be analyzed using computational methods. This shift from traditional microscopy to digital platforms has paved the way for more efficient and objective assessments, reducing the dependence on subjective human interpretations. Even with progress in digital pathology, various obstacles remain in the precise evaluation of breast cancer histopathological images. The intricate nature of tissue formations, differences in staining methods, and the extensive amount of data produced present considerable challenges. Furthermore, the subjective aspect of conventional pathology assessments can lead to variability among observers, affecting the uniformity and dependability of diagnostic conclusions.

The need for rapid yet accurate results in clinical environments calls for expedited image analysis techniques without sacrificing precision. Consequently, there’s an increasing demand for automated and standardized methods in histopathological image classification. Machine learning (ML) and artificial intelligence (AI) are emerging as vital tools in this realm, offering swift, uniform, and unbiased analysis of images. ML and AI have become prominent in breast cancer diagnosis, especially in the analysis of histopathological images. These technologies can enhance the abilities of pathologists, refine diagnostic accuracy, and streamline the analysis workflow. ML algorithms, when trained on extensive datasets of labeled histopathological images, can identify patterns and features related to various types and stages of breast cancer. Once proficient, these algorithms can categorize new images, potentially surpassing traditional methods in consistency and performance. AI systems are also adept at recognizing subtle patterns and variations that might be difficult for human experts to notice.

The challenge in breast cancer diagnosis is the identification of its various histological subtypes, each requiring accurate classification for effective personalized treatment plans. Traditionally, pathologists manually review these images, a process that can be lengthy and subjective. Implementing machine learning and deep learning approaches to automate this task promises quicker, more precise diagnoses, potentially transforming the field of histopathology and improving patient outcomes.

As advancements in breast cancer diagnosis continue to evolve, it is imperative to address accessibility concerns in medical image interpretation, particularly for individuals with disabilities and the elderly. This paper presents a novel framework that modifies the EfficientNetV2-S architecture and integrates a cyclic learning rate strategy to optimize breast cancer histopathological image classification. By tailoring our approach to enhance interpretability, we seek to bridge gaps in accessibility, fostering inclusivity in the understanding and utilization of diagnostic information among diverse demographic groups, including those with disabilities and the elderly.

### 1.1 Objectives of the proposed study

The objective of this study is to develop a precise and efficient machine learning model for the automated classification of breast cancer histopathological images. Specifically, the goals are as follows:

- Implementing an advanced machine learning model based on EfficientNetV2 architecture to achieve high accuracy in classifying benign and malignant tumors.
- Addressing challenges related to generalizability, computational performance, and dataset imbalances encountered in existing models.
- Enhancing model transparency and interpretability to facilitate its applicability in medical diagnostic settings.
- Conducting rigorous training and validation using the BreakHis dataset to evaluate the performance of the proposed model.
- Assessing the model’s efficacy through metrics such as accuracy, precision, recall, F1 score, and Cohen’s Kappa value to demonstrate its superiority over existing methodologies.

### 1.2 Organization of the document

The structure of the remainder of this paper is as follows:

- Literature Review: In the subsequent section, we explore the existing body of research on breast cancer histopathological image classification, emphasizing the advantages and drawbacks of current methods.
- Methodology: Here, we describe our proposed deep learning model, elaborating on its architecture, the preprocessing of data, and the training process of the model.
- Experimental Results: This part details the outcomes of our experiments, demonstrating the enhanced performance of our model through both quantitative assessments and comparisons with existing methods.
- Discussion: In this segment, we analyze the implications of our results, focusing on the clinical relevance of precise classification of breast cancer histopathological images.
- Conclusion: The concluding section summarizes our research contributions and outlines the potential influence of our work on the diagnosis and treatment of breast cancer.

## 2 Literature review

The precise categorization of breast cancer histopathological images holds critical significance in clinical settings, as it directly impacts the diagnosis and treatment planning. There has been notable advancement over time in devising computational methods for the analysis of breast cancer images. This literature review delves into the development of these methodologies, underscoring both their strengths and their limitations.

### 2.1 Traditional approaches

Traditionally, the diagnosis of breast cancer relied on manual examination of histopathological images by experienced pathologists. While pathologists possess extensive expertise, this approach is time-consuming, subject to interobserver variability, and can be prone to errors, especially in distinguishing between subtle histological subtypes (1). The introduction of traditional machine learning approaches marked the beginning of automated image analysis. Characteristics like texture, shape, and color were extracted from histopathological images for the purpose of classification. Techniques such as Support Vector Machines (SVMs), Random Forests, and k-Nearest Neighbors (k-NN) were commonly employed for classification tasks. Although these methods enhanced efficiency, they faced difficulties in dealing with the complexity and variety present in breast cancer images, making accurate classification a persistent challenge (2).

### 2.2 Deep learning revolution

The rise of deep learning, especially Convolutional Neural Networks (CNNs), significantly transformed the realm of breast cancer histopathological image analysis. Deep learning models showed exceptional proficiency in tasks related to image classification, and their application in breast cancer research was a notable development.

- AlexNet and VGG: Early CNN architectures like AlexNet and VGG were adapted for breast cancer image classification. These models, pre-trained on large image datasets, showed promise in achieving higher accuracy compared to traditional methods. However, they struggled with handling diverse histological subtypes and required substantial computational resources (3).
- Transfer Learning: The adoption of transfer learning, involving the fine-tuning of pre-trained CNN models for breast cancer classification, became increasingly prevalent. This approach enabled the utilization of features learned from general image datasets, such as ImageNet, and their adaptation for specialized tasks. Models such as Inception and ResNet were broadly embraced for this purpose (4).

### 2.3 Challenges in deep learning

While deep learning models demonstrated significant improvements in breast cancer image classification, several challenges persisted:

- Diverse Histological Subtypes: Breast cancer encompasses various histological subtypes, each with distinct characteristics. Existing models struggled to accurately differentiate between these subtypes, potentially leading to misclassification (5).
- Imbalanced Datasets: Imbalanced datasets, where certain classes are underrepresented, posed challenges in model training. Models tended to favor the majority class, impacting overall accuracy (6).
- Overfitting: A prevalent challenge in deep learning, overfitting arises when models excel on training data but underperform on new, unseen data. Implementing strategies to counteract overfitting is crucial for the effectiveness of these models (7).
- Generalization: Ensuring that models could generalize to diverse image types, including different magnifying factors, was crucial for real-world applications (8).

### 2.4 Recent advances

- Data Augmentation: To tackle the issues of overfitting and generalization, data augmentation methods were applied. These techniques artificially enlarged the dataset through the application of random transformations to existing images, including rotations, flips, and adjustments in brightness. Data augmentation contributed to enhancing the robustness of the models.
- Attention Mechanisms: Attention mechanisms were introduced to enhance the discriminative capabilities of models. Attention modules allowed models to focus on relevant image regions, improving the accuracy of subtype classification (9).
- Ensemble Learning: Ensemble learning techniques, where multiple models were combined to make predictions, further improved accuracy. Ensemble methods reduced the impact of model bias and variance, leading to more reliable results (9).

In Table 1 different models with respect to their state of art technologies used is explored and a comprehensive survey is conducted.

**Table 1:** Different existing studies.

Study	Objective	Summary
Jafarzadeh Ghoushchi et al. (10)	Advance breast cancer detection via new machine learning with modified deep learning, focusing on CNNs for tumor localization in breast cancer, using BCDRD01 database data.	Introduces a novel machine learning model for early and precise breast cancer diagnosis using VGG-based CNNs for effective image segmentation, demonstrating CNNs’ efficacy in lesion identification.
Humayun et al. (11)	Develop a deep learning model for breast cancer risk prediction using transfer learning with InceptionResNetV2, focusing on gene expression and imaging.	Proposes a model using InceptionResNetV2 with 91% accuracy on breast cancer dataset, highlighting deep learning’s role in risk assessment and automating imaging techniques.
Abunasser et al. (12)	Enhance early detection and classification of breast cancer using deep learning, presenting BCCNN for classifying breast cancers into eight types.	Proposes BCCNN for detection and classification into eight categories, showing a 98.28% F1-score accuracy using MRI images, enhancing diagnosis and patient outcomes.
Ding et al. (13)	Address large-scale, annotated dataset creation for computer-vision models in pathology by introducing SNOW, a synthetic pathological image dataset.	Presents SNOW, a synthetic dataset with 20 k image tiles and over 1.4 million annotated nuclei, effective in training scenarios, demonstrating synthetic data benefits in computational pathology.
Dammu et al. (14)	Use CNNs to predict PCR, RCB, and PFS in breast cancer patients undergoing chemotherapy, focusing on combining MRI and demographic data.	Evaluates three CNN approaches, with the Integrated approach outperforming others. Shows utility of deep learning with MRI in predicting key outcomes, aiding treatment decisions.
Hekal et al. (15)	Introduce an ensemble deep learning system for early breast cancer detection, focusing on SNRs and small nodules, combining CNNs and SVMs.	The ensemble system achieves 94% accuracy in M vs. B classification and 95% in MM vs. BM, showing efficacy on the CBIS-DDSM dataset and outperforming related methods.
Tekin et al. (16)	Introduce Tubule-U-Net for tubule segmentation in breast cancer WSIs, aiming to automate tubule index assessment using a novel dataset.	Tubule-U-Net, using various architectures, excels in performance, particularly with the EfficientNetB3 model, achieving high scores in dice, recall, and specificity on test data.
Obayya et al. (17)	Improve breast cancer classification using an arithmetic optimization algorithm combined with deep learning (AOADL-HBCC), enhancing image classification in healthcare decision-making.	AOADL-HBCC combines median filtering, contrast enhancement, and a SqueezeNet model, achieving 96.77% accuracy, superior to other methodologies.
Raza et al. (18)	Propose DeepBraestCancerNet for accurate breast cancer detection and classification, leveraging recent deep learning advances.	DeepBraestCancerNet achieves 99.35% accuracy, outperforming other models and validating effectiveness with another dataset, reaching 99.63% accuracy.
Jabeen et al. (19)	Address manual breast cancer diagnosis challenges by proposing an automated framework enhancing mammograms, using deep transfer learning and machine learning classifiers.	The framework achieves 95.4 and 99.7% accuracy on CBIS-DDSM and INbreast datasets, surpassing current technologies.

Recent studies have showcased significant progress in utilizing machine learning and deep learning for various aspects of breast cancer diagnosis and treatment. Researchers have developed novel models for early detection and precise localization of tumors using convolutional neural networks (CNNs) on databases like BCDRD01. Transfer learning with architectures like InceptionResNetV2 has been effective in predicting breast cancer risk based on gene expression and imaging data. Another model, BCCNN, demonstrates high accuracy in classifying breast cancer types using MRI images, enhancing diagnostic capabilities. Additionally, the creation of synthetic datasets like SNOW has proven beneficial for training computer-vision models in pathology. One prevalent trend is the widespread adoption of deep learning techniques, particularly convolutional neural networks (CNNs), for automated analysis and classification of histopathological images. These deep learning models, often built upon architectures like EfficientNet, demonstrate remarkable performance in accurately identifying and distinguishing between benign and malignant tissue structures. Additionally, many studies emphasize the importance of advanced preprocessing and augmentation techniques to enhance the quality and diversity of training data, thereby improving model generalization and robustness. Furthermore, the integration of interpretability methods, such as attention mechanisms or feature visualization techniques, enhances the transparency and clinical utility of automated diagnostic systems. Overall, the collective efforts showcased in these studies underscore the significant advancements in breast cancer histopathological image analysis, paving the way for more accurate, efficient, and scalable diagnostic solutions in clinical practice.

## 3 Methodology

**Figure 1:**
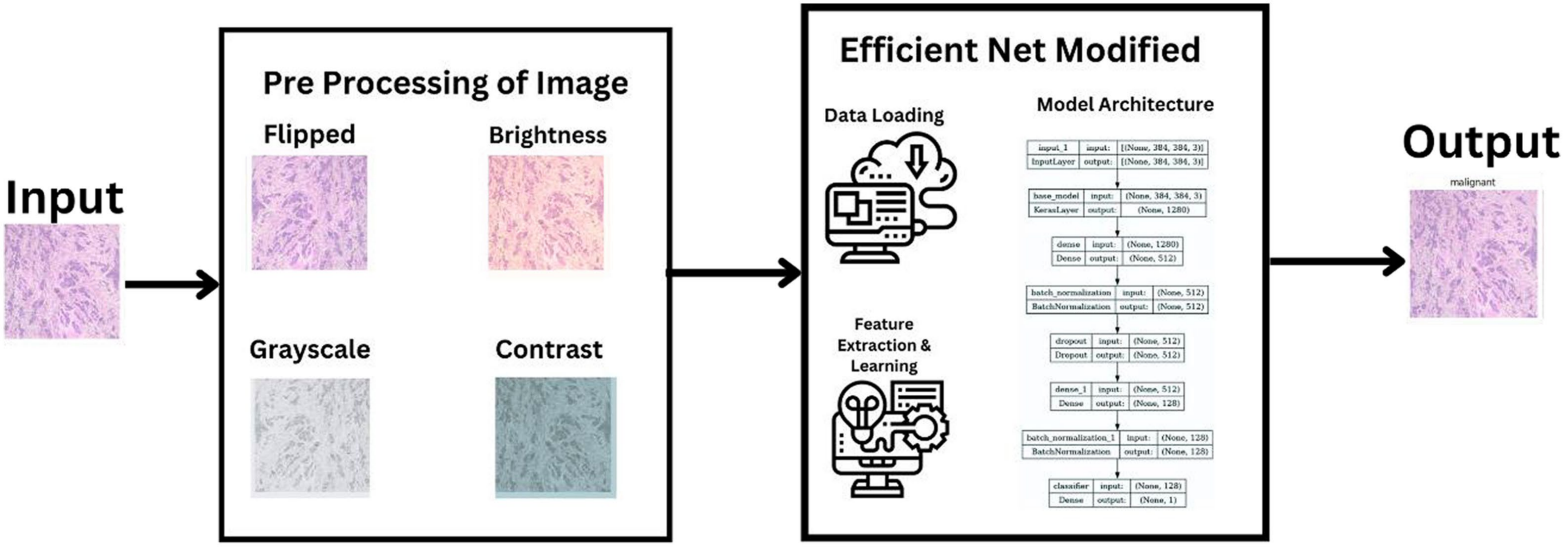
Brief model architecture.

In our research, we adopted a systematic approach to categorize histopathological images of breast cancer, leveraging cutting-edge machine learning techniques. Our process utilized the extensive BreakHis dataset and included stages of data preprocessing, choosing the right model, training it, and then evaluating its performance. We selected the latest neural network frameworks, focusing on different versions of EfficientNetV2. To guarantee a sturdy and precise classification of benign and malignant breast cancer tissues, we employed comprehensive data augmentation and meticulous training methodologies. This section details the entire procedure and methods employed, ranging from the description of the dataset to the application of machine learning models and the metrics used for evaluation. Figure 1 illustrates a summarized architecture of our model which contains multiple pre processing steps along with the model architecture.

### 3.1 Dataset description

The BreakHis dataset stands as a pivotal resource in medical imaging for breast cancer research. Comprising 9,109 microscopic images from 82 patients, it offers a profound look at breast tumor tissues. These images, captured at various magnifications (40×, 100×, 200×, and 400×), reveal intricate histopathological details. The dataset includes 2,480 benign and 5,429 malignant samples, each being a 700×460 pixel, 3-channel RGB image with 8-bit depth per channel, stored in PNG format.

**Figure 2:**
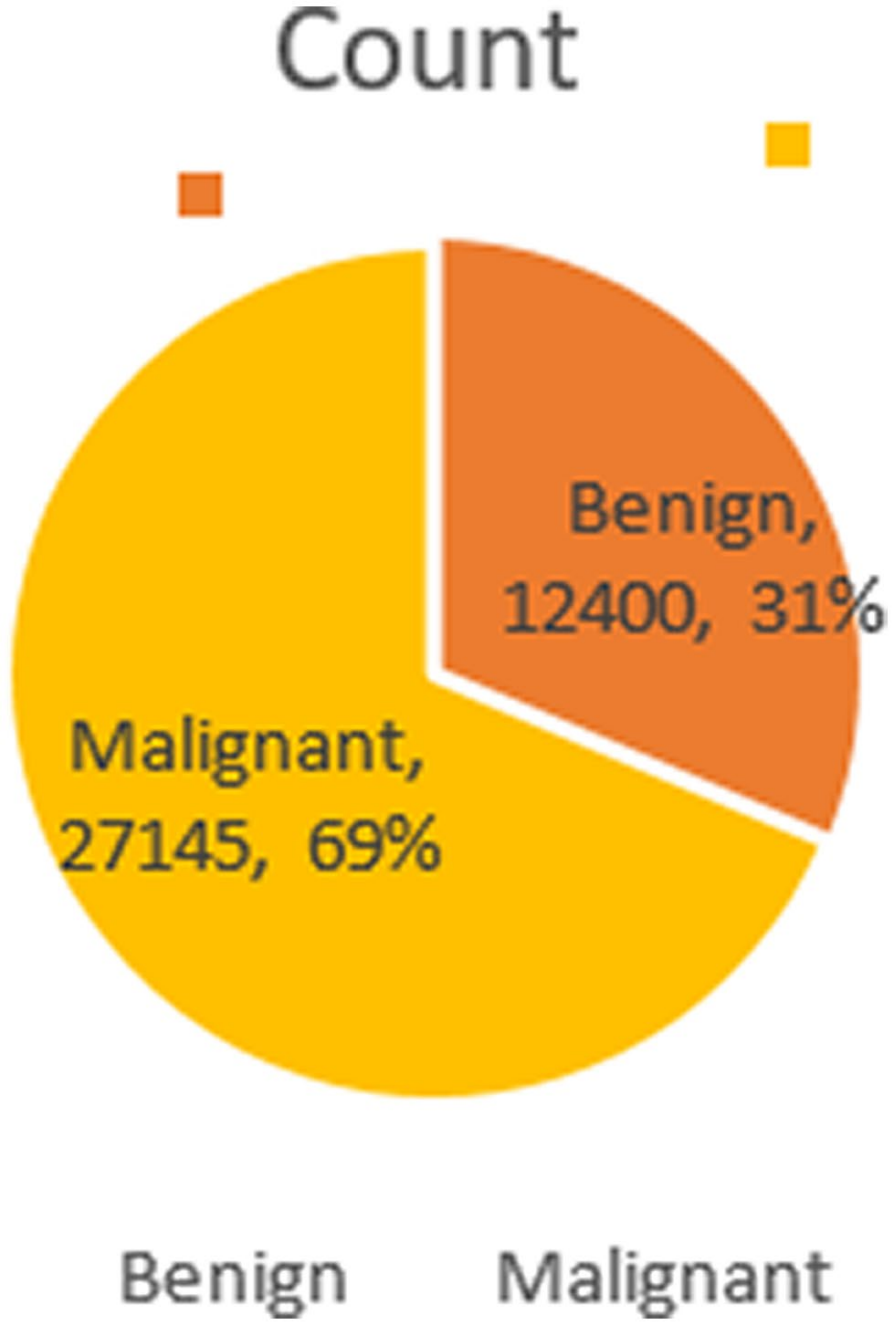
Dataset count.

**Figure 3:**
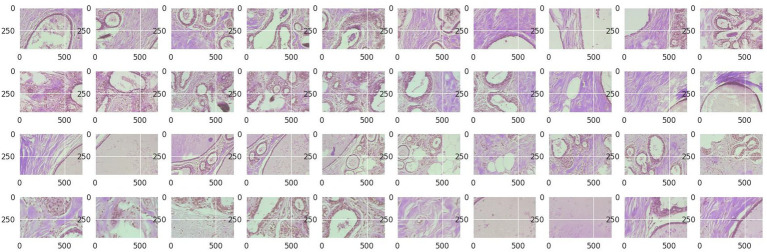
Benign sample images.

**Figure 4:**
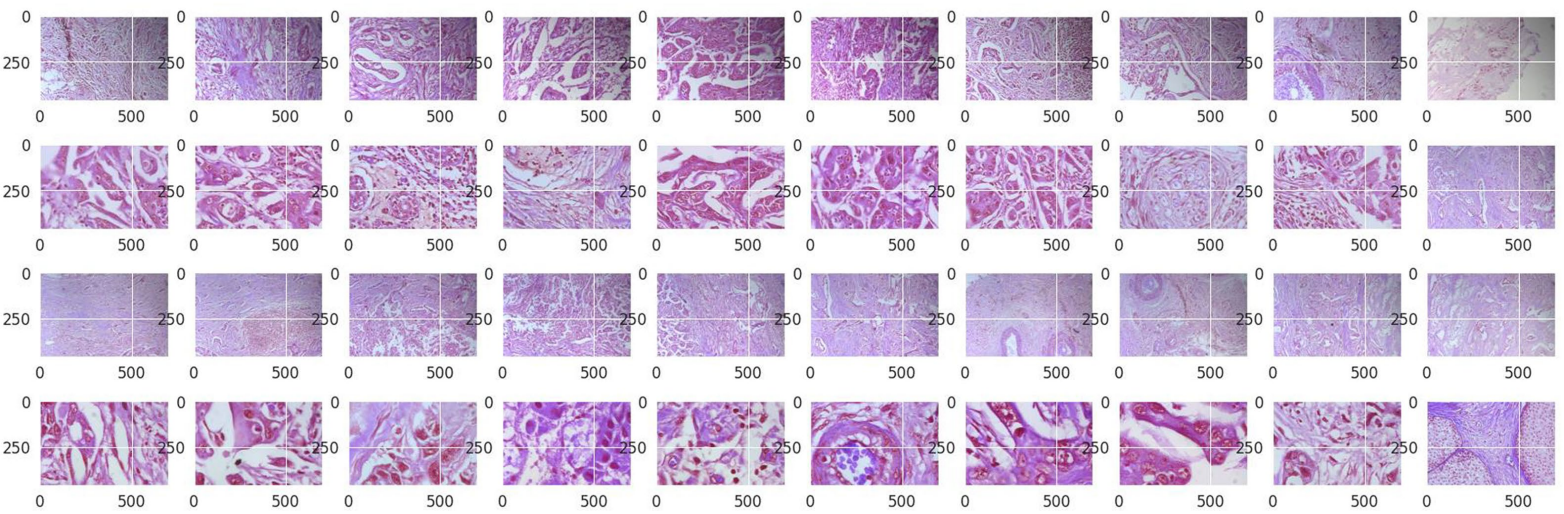
Malignant sample images.

Structured into two principal categories, benign and malignant tumors, the dataset further subdivides into specific tumor types. For benign tumors, it includes adenosis (A), fibroadenoma (F), phyllodes tumor (PT), and tubular adenoma (TA). The malignant tumors comprise ductal carcinoma (DC), lobular carcinoma (LC), mucinous carcinoma (MC), and papillary carcinoma (PC). Each image file is descriptively named, detailing biopsy method, tumor class, type, patient ID, and magnification factor, making it a valuable dataset for automated classification and analysis. Figure 2 visually represents this data distribution while Figures 3, 4 show some Benign and Malignant samples images from the dataset.

The BreakHis dataset, developed in partnership with the P&D Laboratory – Pathological Anatomy and Cytopathology in Paraná, Brazil, holds significant value for its ability to facilitate benchmarking and comparative research in the field of automated cancer diagnosis and histopathological image analysis. This collaboration has resulted in a dataset that is not only extensive but also deeply informative, making it an essential tool for advancements in medical imaging and machine learning applications in breast cancer research.

### 3.2 Preprocessing

The preprocessing of the BreakHis dataset was a vital phase in preparing the images for efficient machine learning analysis. This procedure incorporated various essential steps to normalize and improve the image data, thereby optimizing the performance of the model. These steps included techniques such as resizing images to a uniform dimension, adjusting brightness and contrast for better clarity, and potentially applying normalization or standardization to align the pixel value distributions. This careful preprocessing ensured that the images were suitably formatted and enhanced for the subsequent stages of machine learning and deep learning analysis.

- Resizing Images: Given the variation in image sizes within the dataset, all images were resized to a uniform dimension. The chosen size was dictated by the requirements of the EfficientNetV2 model variants used in the study. Specifically, images were resized to dimensions that matched the input size of the selected EfficientNetV2 model. For instance, by using EfficientNetV2-S, images were resized to 384×384 pixels.
- Normalization: To ensure that the model received input data within a consistent range, normalization of the images was performed. This step involved scaling the pixel values in each image from their original 8-bit range (0–255) to a floating-point range between 0 and 1. This normalization helps in speeding up the training process and improving model convergence.
- Color Consistency: As the images were in RGB format, no color conversion was necessary. However, we ensured that the color consistency was maintained across all images to prevent any color-related biases during model training.
- Data Augmentation: In order to enhance the variety within the training dataset and mitigate the risk of overfitting, we implemented data augmentation techniques. These techniques encompassed random horizontal flipping of images, rotations, adjustments in brightness and contrast, and random resized cropping. This augmentation process was dynamically integrated during the training phase, effectively expanding the dataset by creating varied versions of each image. Such augmentation not only diversified the training data but also helped the model in learning to generalize better from the augmented images, crucial for robust machine learning models.
- Dataset Splitting: The BreakHis dataset was systematically partitioned into training, validation, and test sets. To maintain a representative distribution of both benign and malignant cases across these sets, a stratified sampling method was utilized. This approach ensured that each set mirrored the overall composition of the dataset in terms of the proportion of benign to malignant cases. Additionally, to counteract any class imbalance, particularly evident in the training set, an upsampling technique was applied. This involved increasing the number of images in the underrepresented class (either benign or malignant) to match the count of the other class. Such a measure was crucial to prevent any bias in the model training process, thereby enhancing the accuracy and reliability of the model in distinguishing between benign and malignant breast cancer tissues.
- Batch Processing: Images were grouped into batches for efficient processing during the training phase. The batch size was determined based on the model’s requirements and the computational resources available.
- Image Parsing and Tensor Conversion: Each image file was read and decoded into a tensor format suitable for input into the TensorFlow-based neural network. This conversion process was essential for the compatibility of image data with the machine learning framework used.

**Figure 5:**
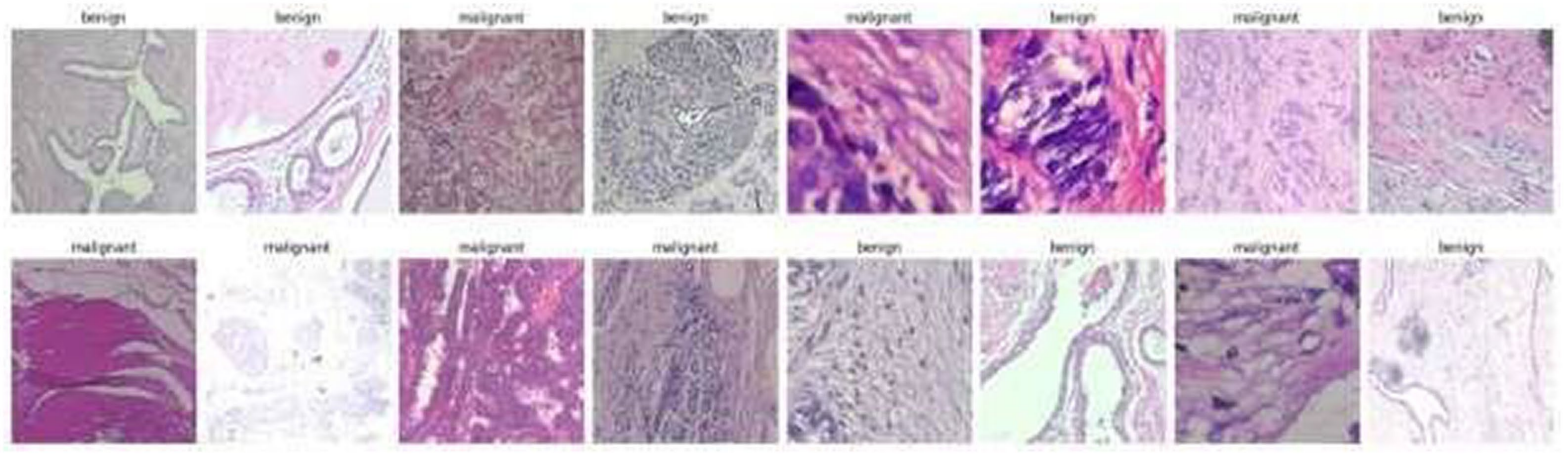
Images from the Break-His dataset.

In Figure 5 some images from the original Break-His dataset is shown.

These preprocessing steps were vital in preparing the BreakHis dataset for the machine learning pipeline, ensuring that the input data was in an optimal format for model training and contributed to the high accuracy and f1 score achieved in the study.

### 3.3 EfficientNetV2 and its variants

The decision to utilize EfficientNetV2 models and their variants in this study was influenced by multiple compelling reasons, closely aligned with our goals of achieving high accuracy and efficiency in the classification of breast cancer histopathological images. EfficientNetV2, an advanced iteration of the original EfficientNet, offers substantial enhancements which render it especially appropriate for this task:

- State-of-the-Art Performance: The EfficientNetV2 models are renowned for their exceptional performance in image classification tasks. They have demonstrated impressive results on benchmark datasets, underscoring their potential for application in medical image analysis where accuracy is of utmost importance. This superior performance is attributed to their advanced architecture, which efficiently balances depth, width, and resolution, leading to highly accurate models. Their proven effectiveness in various image classification challenges makes them a promising option for the intricate task of classifying histopathological images in breast cancer research, where precision in diagnosis is critical.
- Scalability and Versatility: The EfficientNetV2 architecture comes in various sizes (small, medium, and large), providing a scalable solution that can be tailored to the computational resources available and the complexity of the task. This flexibility allows for choosing a model variant that balances between computational efficiency and classification performance.
- Improved Training Speed and Efficiency: EfficientNetV2 models are designed for faster training and higher efficiency compared to their predecessors. This is achieved through optimizations such as Fused-MBConv layers and a more efficient scaling method. For a dataset as large and complex as BreakHis, these improvements in training speed are crucial for timely model development and iteration.
- Advanced Regularization Techniques: These models incorporate the latest advancements in regularization and data augmentation strategies, such as progressive learning and model regularization methods. This is particularly beneficial in a medical imaging context, where the model needs to generalize well from limited and varied training data, reducing the risk of overfitting.
- Optimized for Diverse Image Resolutions: EfficientNetV2 is designed to adeptly manage a range of image resolutions. This trait is particularly important in the analysis of histopathological images, where recognizing details at multiple scales is essential for precise tumor categorization. Its capability to consistently deliver strong performance at various magnifications, such as 40×, 100×, 200×, and 400× as demonstrated in the BreakHis dataset, without requiring substantial changes to the architecture, stands out as a notable benefit.
- Robustness to Variations in Input Data: Given the inherent variations in medical images due to differences in staining techniques, tissue preparation, and imaging conditions, the robustness of EfficientNetV2 models to such variations makes them an apt choice for this application.
- Proven Effectiveness in Medical Imaging: EfficientNet architectures have shown remarkable success in a variety of medical imaging applications, showcasing their proficiency in processing intricate image data and identifying pertinent features for precise disease identification.
- Compatibility with Transfer Learning: The availability of pre-trained EfficientNetV2 models on standard image datasets like ImageNet allows for leveraging transfer learning. This approach is particularly useful in medical imaging tasks, where annotated data can be scarce. Pre-trained models can be fine-tuned on the specific medical dataset, significantly improving the learning process.

In the neural network structure developed for categorizing breast cancer histopathological images, every layer is carefully crafted to assist in the precise distinction between benign and malignant tumors. The architecture begins with the EfficientNetV2 as the base model, acting as a sophisticated feature extractor. This model outputs a 1,280-dimensional feature vector for each image and contains over 20 million trainable parameters, reflecting its capability to learn complex patterns in the image data. Following this, a dense layer compresses these features from 1,280 to 512 dimensions, allowing for a more compact yet informative representation of the image characteristics.

To enhance the training process and prevent overfitting, a batch normalization layer follows, normalizing the output of the dense layer by scaling and shifting. This is crucial for stabilizing learning and maintaining consistency across batches of data. A dropout layer is then employed, which randomly zeroes a fraction of the input units, further aiding in generalization and preventing the model from becoming overly reliant on specific features. The convolution process is depicted in Eq. 1, succeeded by the cyclic learning rate shown in Eq. 2, and the binary cross-entropy loss function is detailed in Eq. 3.


(1) $$\left(I*K\right)\left(i,j\right)=\underset{m}{\sum }\underset{n}{\sum }I\left(m,n\right)K\left(i-m,j-n\right)$$

This equation illustrates the convolution process, where “*I*” denotes the input image and “*K*”: represents the kernel or filter. Convolution is a core operation in CNN architectures, including EfficientNet.


(2) $$\begin{array}{c} CLR={LR}_{\text{min}}+\frac{1}{2}\left({LR}_{\text{max}}-{LR}_{\text{min}}\right)\\ \times \left(1+\cos\left(\frac{\mathrm{epoch}}{\mathrm{total}\;\mathrm{epoch}}\times \pi \right)\right) \end{array}$$

(3) $$BCE\;\mathrm{Loss}=-\frac{1}{N}\overset{N}{\underset{i=1}{\sum }}\left[y_{i}\log\left(p_{i}\right)+\left(1-y_{i}\right)\log\left(1-p_{i}\right)\right]$$

In Table 2 a complete description of the layers is given and in Table 3 the detailed algorithm is given.

**Table 2:** Description of model architecture.

Layer (type)	Output Shape	Param #
base_model (KerasLayer)	(None, 1,280)	20,331,360
dense (Dense)	(None, 512)	655,872
batch_normalization (BatchNormalization)	(None, 512)	2048
dropout (Dropout)	(None, 512)	0
dense_1 (Dense)	(None, 128)	65,664
batch_normalization_1 (BatchNormalization)	(None, 128)	512
classifier (Dense)	(None, 1)	129

**Table 3:** Algorithm of breast cancer image classification.

**Initialization:** Define model handles and image sizes for EfficientNetV2-S-21 k-ft1k and related libraries. **Data Preparation:** Load the breast cancer image dataset from the provided path. Split the dataset into training, validation, and test sets. Address class imbalance by upsampling the minority class. **Data Augmentation:** Implement data augmentation techniques, including horizontal flip, rotation, brightness contrast adjustment, and random crop. **Neural Network Construction:** Choose EfficientNetV2-S-21 k-ft1k as the pre-trained neural network model. Extend the model with custom layers for binary classification (benign/malignant). **Training Configuration:** Set the batch size, number of epochs, and a cyclical learning rate schedule. Compile the model using binary cross-entropy loss and relevant evaluation metrics. **Training the Model:** Train the model on the augmented training dataset. Save the best model based on validation performance. **Training Evaluation:** Visualize training and validation accuracy/loss curves. **Test Set Evaluation:** Evaluate the model on the test set. Display classification metrics such as precision, recall, F1 score, and accuracy. Visualize the confusion matrix to assess model performance.

The network’s subsequent layers include another dense layer, which further compresses the feature vector to 128 dimensions, and a second batch normalization layer, continuing the process of feature refinement and stabilization. The final layer, a classifier, is a dense layer with a single neuron, responsible for making the final binary decision—classifying the tumor as benign or malignant. This layer’s output represents the culmination of a complex process of feature extraction, compression, and refinement, leveraging the intricate patterns learned from the histopathological images to arrive at a precise classification.

Choosing EfficientNetV2 models for the classification of breast cancer histopathological images is supported by their exceptional combination of efficiency, scalability, and cutting-edge performance. Their ability to handle diverse image resolutions and robustness to input variations aligns well with the challenges presented by the BreakHis dataset, ultimately contributing to the high accuracy and f1 scores achieved in this study.

### 3.4 Training process

The development of the breast cancer histopathological image classification model was rigorously planned to maximize performance. We commenced with data augmentation, a critical step in enhancing the model’s generalization capabilities. This process included applying transformations such as horizontal flipping, rotation, random brightness and contrast adjustments, and random resized cropping directly during training. This on-the-fly augmentation introduced a beneficial variability to the dataset, aiding the model in learning to identify tumors under different conditions.

Central to our training strategy was the implementation of a Cyclical Learning Rate (CLR). This technique involved dynamically varying the learning rate between a lower and an upper bound throughout the training epochs. Starting from a relatively high initial rate, it was adjusted to reach a peak and then cycled back, allowing the model to effectively navigate the loss landscape and avoid getting trapped in local minima.

The model training was conducted in batches, a decision driven by the need for efficient memory utilization and the advantages of parallel processing. Batch sizes were carefully chosen to balance the computational load and the model’s complexity. Additionally, batch processing complemented the effectiveness of batch normalization layers by normalizing activations across each batch.

In terms of model compilation, we chose the Binary Crossentropy loss function, suitable for our binary classification task, and paired it with a Stochastic Gradient Descent (SGD) optimizer. This optimizer was integrated with the CLR scheduler to facilitate dynamic learning rate adjustments. Using the SGD optimizer ensured steady and effective convergence during training.

**Figure 6:**
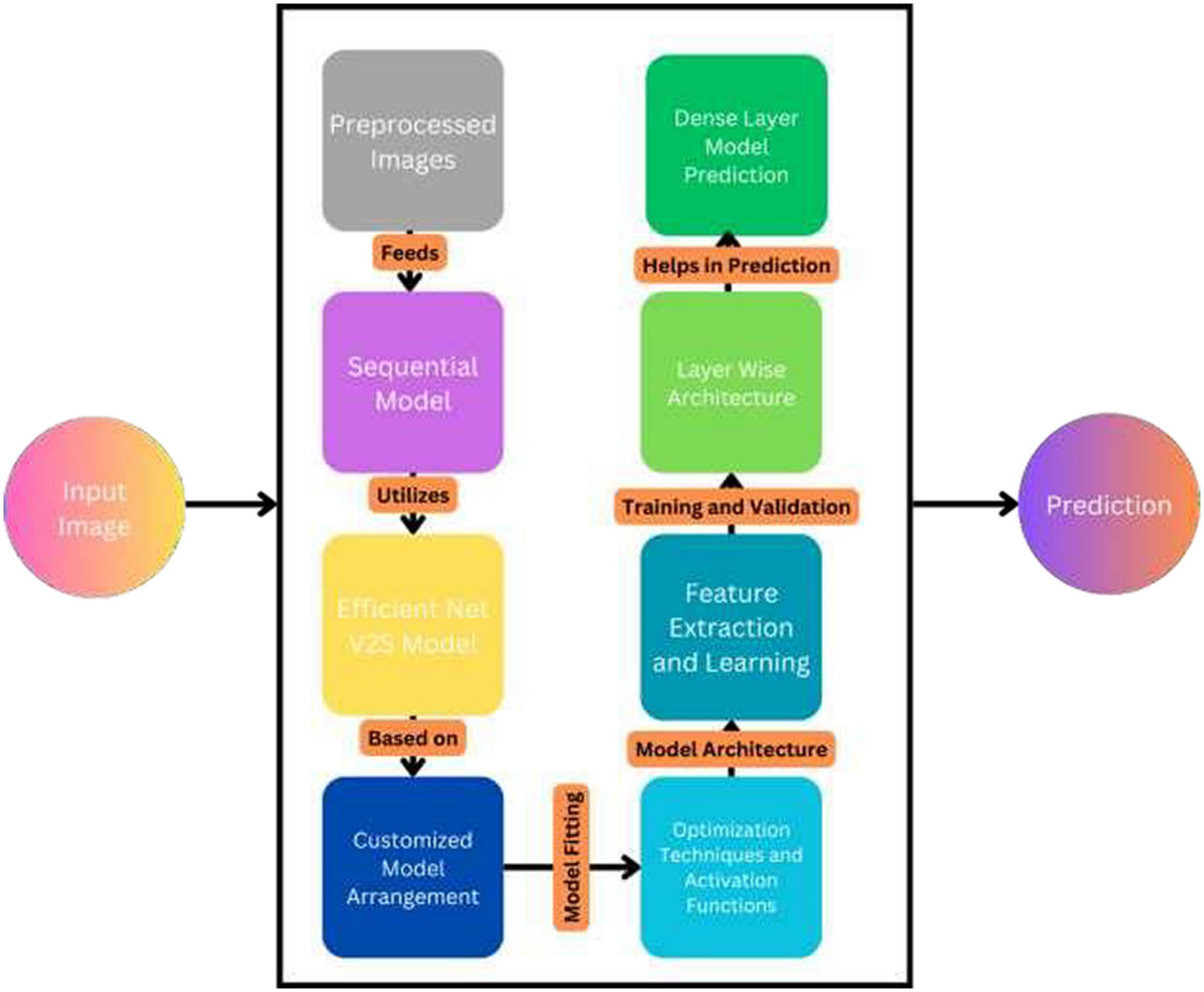
Outline of the process.

The fitting of the model was a crucial phase, where we trained it using the prepared dataset, regularly evaluating its performance on a separate validation set. This phase also included the use of callbacks like ModelCheckpoint to save the best-performing model iteration, ensuring that we retained the model version with the highest validation accuracy. In Figure 6 an outline of the whole training process is given.

During the model’s training phase for classifying breast cancer histopathological images, vital metrics like accuracy, precision, recall, and the F1 score were meticulously tracked after each training epoch. These indicators were crucial in evaluating the model’s effectiveness and informed decisions on modifying its architecture, learning rates, or augmentation methods in further training cycles. This thorough, iterative process was key to crafting an efficient and robust model capable of accurately and reliably classifying breast cancer images. The training approach’s cornerstone was a blend of dynamic learning rate modification, strategic data augmentation, and careful batch processing. These components collectively ensured that the model not only achieved high performance metrics but also remained efficient in terms of training duration and resource consumption, establishing it as an effective instrument for medical image analysis in breast cancer detection.

### 3.5 Model evaluation metrics

For the assessment of our model dedicated to classifying breast cancer histopathological images, we utilized a variety of metrics to thoroughly evaluate its performance. These measures are essential for comprehensively gauging the model’s efficacy and dependability. The key metrics used were:

- Accuracy: Accuracy, a straightforward performance metric, is the ratio of correctly predicted cases to the total number of observations (20). It provides a fundamental insight into the frequency of correct predictions made by the model, regardless of class distribution. In binary classification tasks like ours, accuracy represents the fraction of accurate outcomes (encompassing both true positives and true negatives) in the total cases evaluated. This metric is determined using Eq. 4, where TP stands for True Positive, TN for True Negative, FP for False Positive, and FN for False Negative.

(4) $$\mathrm{Accuracy}=\frac{TP+TN}{TP+TN+FP+FN}$$

- Precision: Precision, also referred to as the positive predictive value, measures the ratio of correctly predicted positive instances to the total predicted positives (21). It addresses the query: Among all the tumors predicted as malignant by the model, how many were truly malignant? High precision corresponds to a low false positive rate. In the field of medical diagnostics, precision is of paramount importance since false positives can result in unwarranted anxiety and medical interventions. It is computed using Eq. 5.

(5) $$\mathrm{Precision}=\frac{TP}{TP+FP}$$

- Recall (Sensitivity): Recall, often referred to as sensitivity, quantifies the ratio of correctly predicted positive instances to all instances within the actual class. It answers the question: Out of all the malignant tumors present, how many did the model accurately detect? In the medical context, achieving a high recall rate is crucial because missing a malignant tumor (false negative) can carry significant consequences for the patient. Recall is computed using Eq. 6.

(6) $$\mathrm{Recall}=\frac{TP}{TP+FN}$$

- F1 Score: The F1 Score serves as the weighted average of Precision and Recall. Consequently, this score considers both false positives and false negatives, offering a balanced assessment of a classifier’s performance (22). It is a valuable metric to demonstrate that a classifier attains high values for both recall and precision. Moreover, the F1 Score is particularly useful when dealing with imbalanced class distributions since it harmonizes the significance of precision and recall. This metric is calculated using Eq. 7.

(7) $$F1\;\mathrm{Score}=2\times \frac{\mathrm{Precision}\times \mathrm{Recall}}{\mathrm{Precision}+\mathrm{Recall}}$$

- Cohen’s Kappa Coefficient: Cohen’s Kappa is a statistical measure that is used to evaluate the accuracy of a classification model while considering the agreement that occurs by chance. It’s particularly useful in scenarios where the class distribution is imbalanced. It is calculated using Eq. 8.

(8) $$k=\frac{p_{o}-p_{e}}{1-p_{e}}$$

- where *po* is the relative observed agreement, and *pe* is the hypothetical probability of chance agreement.

**Figure 7:**
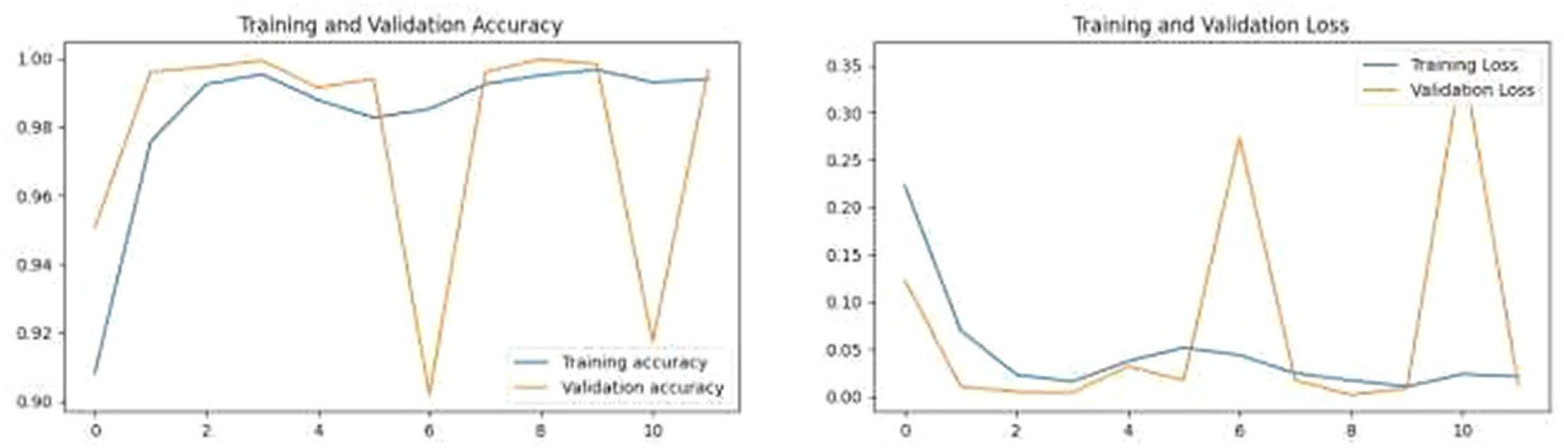
Training and validation accuracy and loss.

These metrics provided a multi-faceted view of the model’s performance. Accuracy gave a quick snapshot of overall effectiveness, while precision and recall offered deeper insights into its ability to correctly identify malignant tumors and minimize false diagnoses. The F1 score played a crucial role as a unified metric that integrated precision and recall, delivering a well-balanced measure of the model’s diagnostic accuracy. This was particularly significant given the uneven distribution of benign and malignant cases within the dataset. The training and validation loss, as well as accuracy, of this model is visualized in Figure 7.

The amalgamation of these metrics provided a comprehensive insight into the model’s capabilities, assuring its reliability and suitability for clinical use in the context of breast cancer diagnosis.

## 4 Experimental results

The outcomes of our research, centered on the classification of histopathological images of breast cancer utilizing the BreakHis dataset, showcased the effectiveness and resilience of the selected machine learning model, EfficientNetV2. The evaluation metrics employed to appraise the model’s performance offered a thorough perspective on its capabilities.

**Figure 8:**
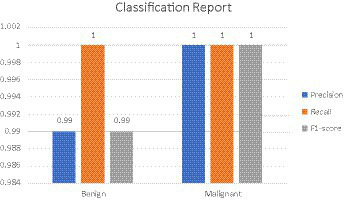
Classification report.

**Figure 9:**
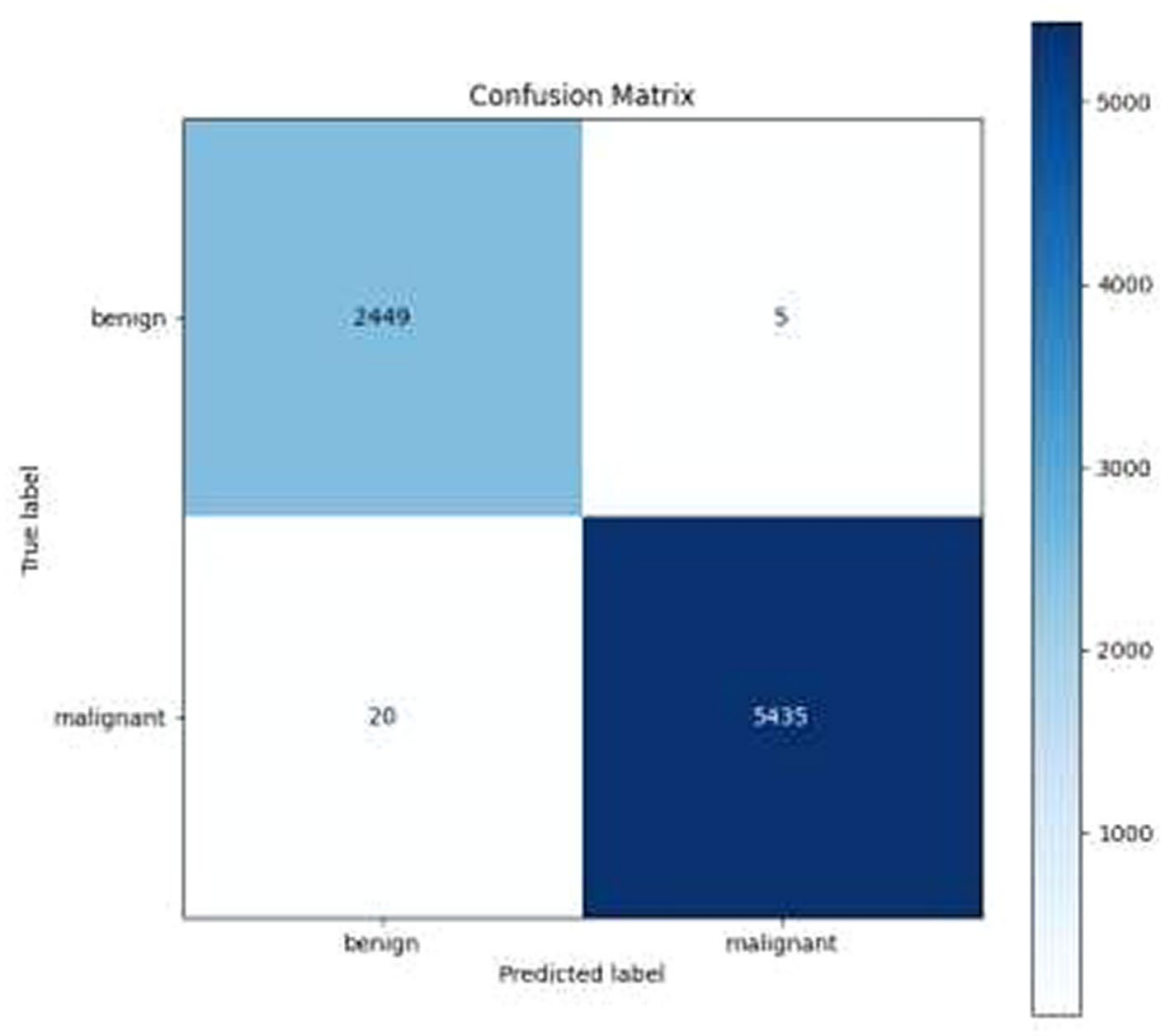
Confusion matrix.

**Figure 10:**
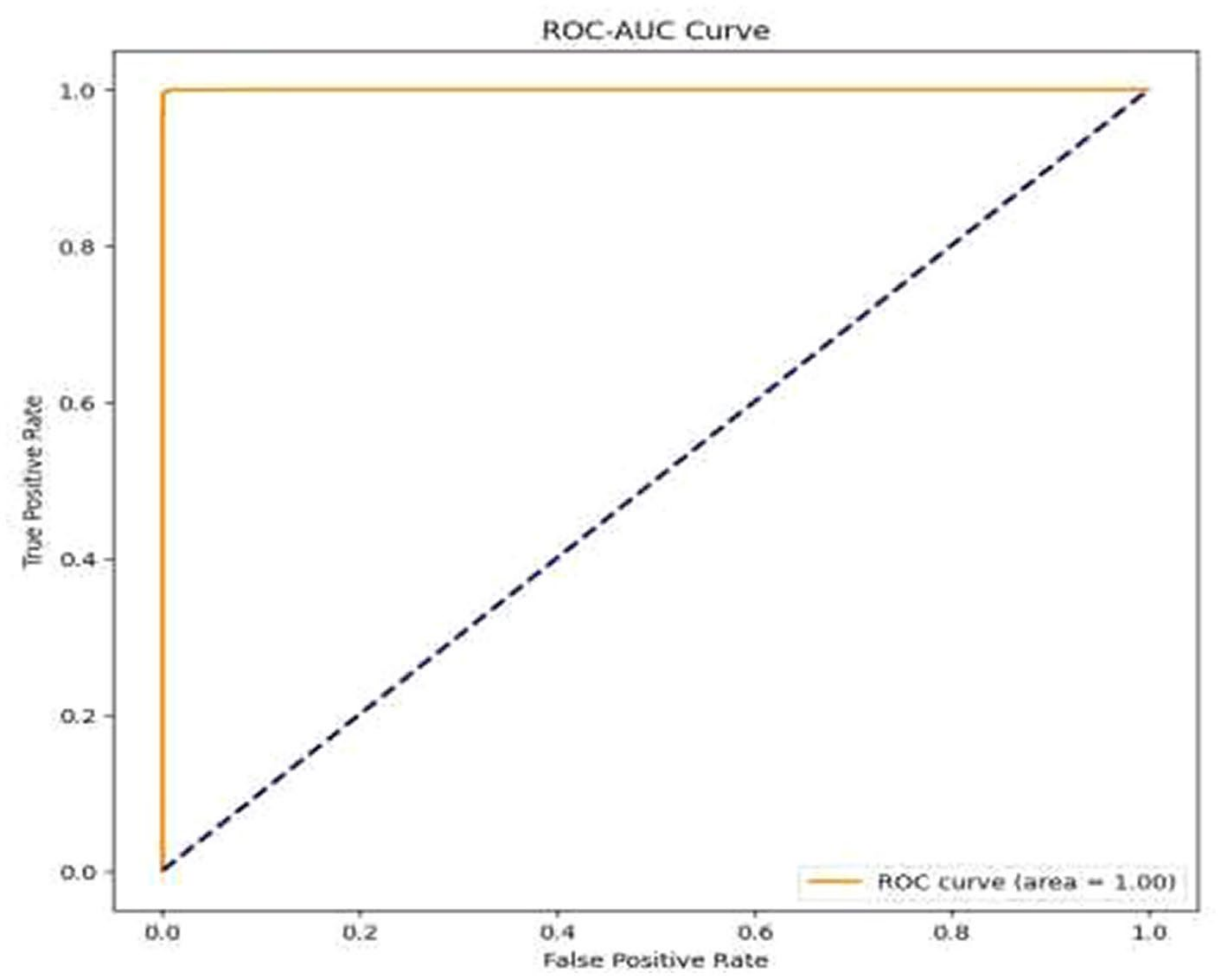
ROC-AUC curve.

**Figure 11:**
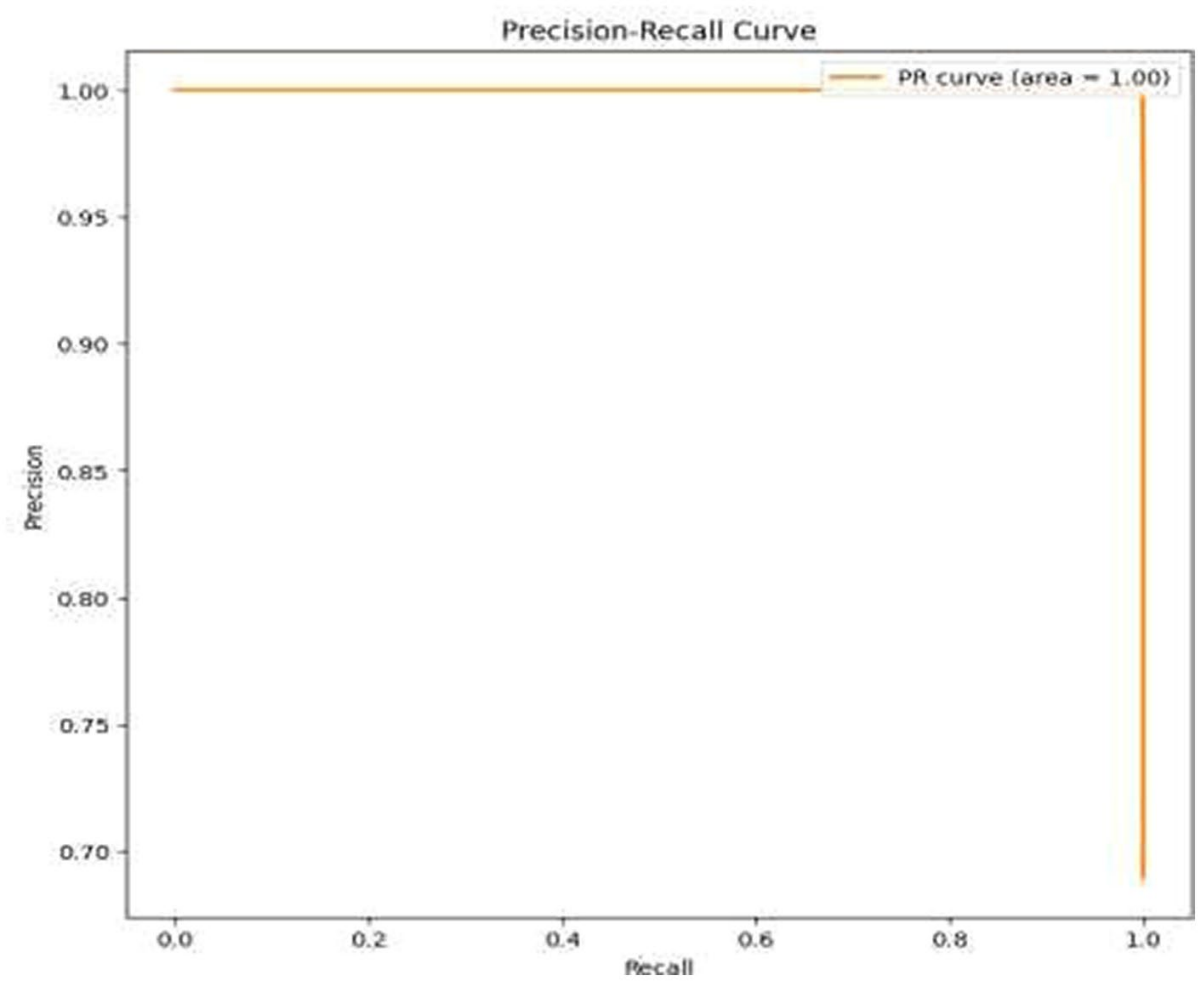
Precision-recall curve.

**Figure 12:**
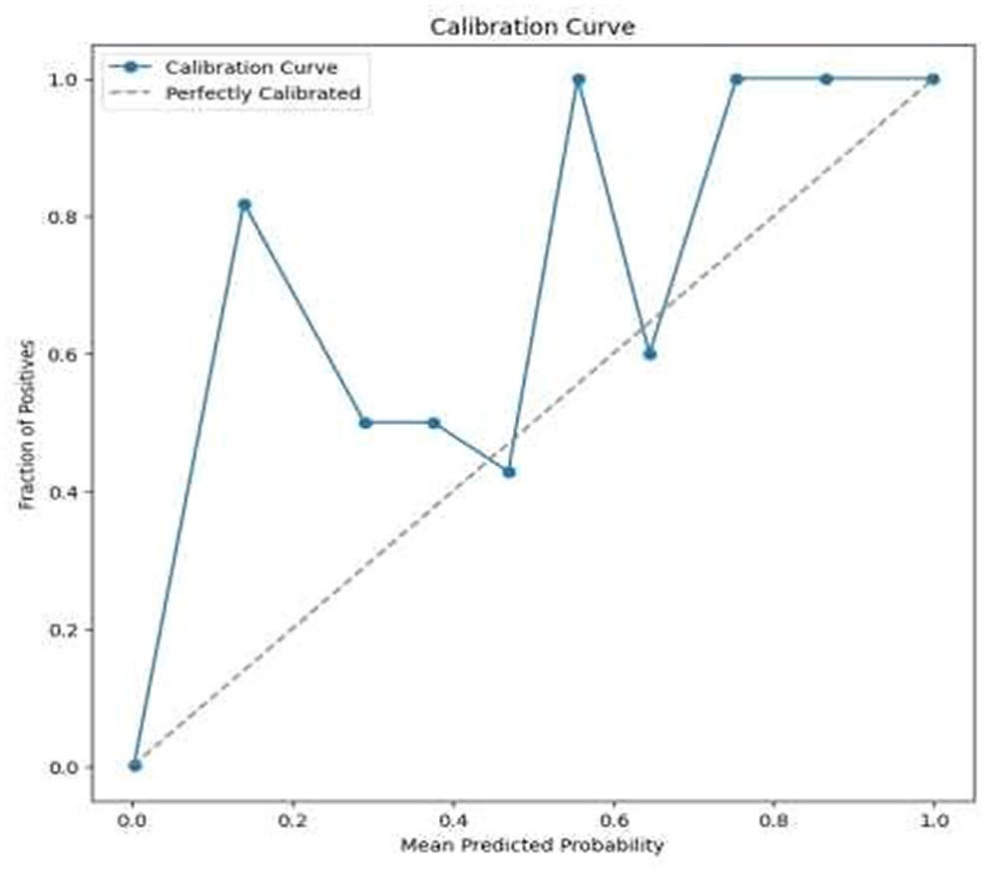
Calibration curve.

- High Accuracy: The model attained an exceptionally high accuracy score of 99.68%. This signifies that the model adeptly categorized the majority of the images as either benign or malignant. Such a remarkable level of accuracy holds great importance in medical diagnostics, where the consequences of misclassification can be substantial.
- Precision and Recall: Both precision and recall metrics further underscored the model’s impressive performance. High precision signified that the model exhibited a low rate of false positives, a critical aspect in medical diagnostics to prevent unwarranted treatment or anxiety. Meanwhile, high recall indicated that the model effectively detected the majority of malignant cases, ensuring timely and appropriate medical intervention when needed.
- F1 Score: The F1 score, serving as a balance between precision and recall, also stood out with a notably high value of 99.77%. This illustrates that the model effectively struck a strong equilibrium between accurately identifying malignant cases (recall) and minimizing false positive diagnoses (precision).
- Cohen’s Kappa: The model achieved a Cohen’s Kappa score of 99.26% indicating high level of binary classification of this model. This high level of agreement, as measured by Cohen’s Kappa, complements the model’s high accuracy, reinforcing its reliability in classifying breast cancer images. Along with high accuracy, precision, recall, and F1 score, the Cohen’s Kappa score further validated the model’s effectiveness. Its ability to account for random chance in the agreement makes it a particularly valuable metric in the context of medical image classification, where the cost of misdiagnosis can be significant.
- Confusion Matrix and Classification Report: The results were additionally reinforced by a comprehensive classification report and a visualized confusion matrix. The classification report furnished insights into the model’s performance across various classes, while the confusion matrix provided a graphical representation of the model’s predictions compared to the actual labels. The classification report is presented in Table 4, followed by visual representations of the classification report in Figure 8 and the confusion matrix in Figure 9.
- Visual Representation of Results: The study also included visual aids such as graphs and charts to illustrate the model’s performance across various metrics. These visualizations helped in better understanding the distribution and nature of the model’s predictions. For the same ROC-AUC Curve along with Precision-Recall Curve is plotted which is been shown in Figures 10, 11.
- Comparison with Baseline Models: The results were compared with baseline models to highlight the improvements and effectiveness of the chosen architecture. This comparison provided a context for the model’s performance, showcasing its superiority in terms of accuracy and reliability over more traditional or less sophisticated models. In Table 5 a brief comparison with existing models is shown.
- Error Analysis: An analysis of the misclassified images was conducted to understand the circumstances under which the model failed. This analysis helped in identifying any patterns in errors, such as specific types of tumors that were consistently misclassified, or issues related to image quality or resolution. For the same calibration curve was plotted which is visualized in Figure 12.
- Robustness to Variations: The model’s robustness was evident in its consistent performance across different magnifications and subtypes of tumors within the dataset. This aspect was crucial, considering the diverse nature of histopathological images in terms of magnification, staining, and tumor types.
- Insights and Observations: The research yielded valuable insights into the model’s decision-making process. For instance, it delved into the regions of the images that garnered the model’s attention during the prediction process, providing insight into which features or patterns held the most significance in the classification procedure.

**Table 4:** Classification report.

	Precision	Recall	F1-score
Benign	0.99	1	0.99
Malignant	1	1	1

**Table 5:** Comparison with existing studies.

Study	Techniques	Accuracy
Jha et al. (23)	Densenet 201	89%
Faris and Badamasi (24)	Convolutional neural network	40%
Rani et al. (25)	Enhanced CNN	93%
Gami et al. (26)	CNN with CAD	82%
Balasubramaniam et al. (27)	LeNet CNN	89.91%
Aidossov et al. (28)	Bayesian network with CNN	90.85%
Nadkarni and Noronha (29)	Inception -V3	90.8%
Sasirekha et al. (30)	Support vector machine	81.1%
Mahmoud Ouf et al. (2023) (31)	CancerNet	86%
Or Rashid et al. (32)	MLP with Adam optimizer	92.44%
Proposed model	EfficientNet V2S modified architecture with cyclic learning rate	99.68%

Overall, the results were highly encouraging, indicating that the model not only achieved exceptional accuracy but also balanced precision and recall effectively. This balance is critical in the medical field, where both over-diagnosis and under-diagnosis carry significant consequences (33, 34). The high F1 score further confirmed the model’s ability to perform consistently well in both respects. These findings highlight the potential of employing advanced machine learning models such as EfficientNetV2 in the realm of medical image analysis, especially for critical tasks like cancer diagnosis.

## 5 Discussion

The outcomes of our study, which centered on the classification of breast cancer histopathological images utilizing the EfficientNetV2 model, offer numerous significant insights into the efficacy and potential applications of machine learning in the field of medical diagnostics.

- High Accuracy and Its Implications: The exceptionally high accuracy (99.68%) achieved by the model is indicative of its robustness and reliability in classifying histopathological images. In the context of medical imaging, such accuracy is paramount, as it directly impacts diagnostic decisions (35). The exceptional level of accuracy achieved by the model implies that it can serve as a valuable tool to aid pathologists, potentially enhancing the efficiency and throughput of diagnostic procedures in clinical settings (36).
- Precision, Recall, and F1 Score: The equilibrium between precision and recall, exemplified by the high F1 score, holds paramount importance in medical diagnostics. Elevated precision reduces the occurrence of false positives, a crucial aspect in avoiding unnecessary medical interventions (37). Simultaneously, a high recall rate ensures that the model accurately identifies the majority of malignant cases, a critical factor in patient care since missed diagnoses can lead to severe consequences (38). The results affirm that the model can effectively sustain this balance, establishing it as a dependable tool in the diagnostic process.
- Analysis of Misclassifications: The error analysis, where misclassified images were examined, provides valuable insights into the model’s limitations. Understanding the characteristics of these misclassified cases can guide future improvements in the model (39). For example, if certain tumor subtypes are consistently misclassified, additional training data or model adjustments targeting these subtypes might be necessary.
- Model Robustness: The consistent performance of the model across various magnifications and tumor types underscores its robustness. This aspect is particularly important given the diverse nature of medical images in real-world settings, where variability in factors like staining techniques and image quality is common (40).
- Clinical Integration and Future Work: While the results are promising, the integration of such models into clinical workflows requires careful consideration. Factors such as model interpretability, integration with existing health IT systems, and adherence to regulatory standards are critical for successful implementation (41). Future endeavors could center on multi-class classification to differentiate between various types of breast cancer, thereby expanding the model’s applicability in clinical diagnostics (42). Furthermore, assessing the model’s performance on external datasets and in real-world clinical environments would be crucial to validate its applicability and robustness further.

This study highlights the substantial potential of advanced machine learning models in the domain of medical image analysis, especially in critical applications like cancer diagnosis. The model’s impressive levels of accuracy, precision, recall, and F1 score not only validate its effectiveness but also underscore the capacity of such technologies to complement and elevate current diagnostic approaches in healthcare (43).

## 6 Conclusion

In this study, we delved into the utilization of the EfficientNetV2 model for the classification of breast cancer histopathological images and obtained promising outcomes. The model demonstrated a remarkable accuracy of 99.68%, a high F1 score, and an excellent balance between precision and recall. These findings suggest that the model has significant potential as an aid in diagnosing breast cancer, potentially enhancing the efficiency and accuracy of pathologists’ work. Nonetheless, it’s important to acknowledge the limitations of this study. The model’s training and evaluation were conducted solely on the BreakHis dataset, which, although extensive, may not encompass the entire spectrum of clinical scenarios. The absence of external validation raises concerns about the model’s ability to generalize to other datasets. Additionally, the inherent “black box” nature of deep learning models, including the one used in this study, presents challenges in terms of interpretability, a critical aspect in the context of medical diagnosis. Also, the inherent data imbalance in the dataset might have influenced the model’s learning and performance.

Looking forward, there are several avenues for further research. Testing the model on external datasets from diverse clinical environments is crucial for assessing its real-world applicability. Enhancing the model’s interpretability would be invaluable in gaining trust and acceptance in clinical settings. Expanding the model to include multi-class classification could provide more detailed diagnostic information, and exploring advanced techniques to handle data imbalance could improve its ability to recognize less common, yet clinically significant cases. Finally, conducting clinical trials would be a critical step in evaluating the model’s practical utility and integration into medical workflows. In conclusion, this study underscores the immense potential of advanced machine learning in the field of medical imaging, especially for crucial tasks such as cancer diagnosis. The high-performance metrics achieved by the model highlight its potential as a reliable diagnostic tool, yet further research and validation are necessary to overcome its limitations and fully harness its capabilities in clinical settings.

## Data availability statement

The original contributions presented in the study are included in the article/supplementary material, further inquiries can be directed to the corresponding author.

## Author contributions

MAM: Conceptualization, Formal analysis¸ Project administration, Writing – review & editing. TM: Conceptualization, Data curation, Software, Writing – original draft. AT: Conceptualization, Data curation, Software, Writing – review & editing. VV: Data curation, Formal analysis, Validation, Visualization, Writing – review & editing. SK: Conceptualization, Methodology, Supervision, Writing – review & editing. MAlo: Formal analysis, Project administration, Visualization¸ Writing – review & editing.
